# Combined BSA-Seq and RNA-Seq Analysis to Identify Candidate Genes Associated with Aluminum Toxicity in Rapeseed (*Brassica napus* L.)

**DOI:** 10.3390/ijms252011190

**Published:** 2024-10-17

**Authors:** Huiwen Zhou, Paolan Yu, Lanhua Wu, Depeng Han, Yang Wu, Wei Zheng, Qinghong Zhou, Xiaojun Xiao

**Affiliations:** 1 Institute of Jiangxi Oil-Tea Camellia, College of Pharmacy and Life Science, Jiujiang University, Jiujiang 332005, China; hwzhou0320@jju.edu.cn (H.Z.); 6090070@jju.edu.cn (L.W.); 6090067@jju.edu.cn (Y.W.); 2 Jiangxi Institute of Red Soil and Germplasm Resources, Key Laboratory of Arable Land Improvement and Quality Improvement of Jiangxi Province, Nanchang 330046, China; 15270904956@163.com (P.Y.); handepeng1113@163.com (D.H.); zw07917043299@163.com (W.Z.); 3 Key Laboratory of Crop Physiology, Ecology and Genetic Breeding, Ministry of Education, Agronomy College, Jiangxi Agricultural University, Nanchang 330045, China

**Keywords:** *Brassica napus*, aluminum resistance, BSA-seq, transcriptome, candidate genes

## Abstract

Exchangeable aluminum (Al) ions released from acidic soils with pH < 5.5 inhibit root elongation of crops, ultimately leading to yield reduced. It is necessary to identify the quantitative trait locus (QTLs) and candidate genes that confer toxicity resistance to understand the mechanism and improve tolerance of rapeseed. In this study, an F_2_ segregating population was derived from a cross between Al-tolerance inbred line FDH188 (R178) and -sensitive inbred line FDH152 (S169), and the F_2:3_ were used as materials to map QTLs associated with the relative elongation of taproot (RET) under Al toxicity stress. Based on bulked segregant analysis sequencing (BSA-seq), three QTLs (*qAT-A07-1*, *qAT-A07-2*, and *qAT-A09-1*) were detected as significantly associated with RET, and 656 candidate genes were screened. By combined BSA and RNA-seq analysis, 55 candidate genes showed differentially expressed, including genes encoding ABC transporter G (ABCG), zinc finger protein, NAC, ethylene-responsive transcription factor (ERF), etc. These genes were probably positive factors in coping with Al toxicity stress in rapeseed. This study provides new insight into exploring the QTLs and candidate genes’ response to Al toxicity stress by combined BSA-seq and RNA-seq and is helpful to further research on the mechanism of Al resistance in rapeseed.

## 1. Introduction

Aluminum (Al) is the most abundant metallic element found in the earth’s crust. When the soil pH value drops below 5.5, exchangeable Al ions (primarily A1^3+^, Al(OH)_2_^+^, and Al(OH)^2+^) released from silicates or oxides can promote exponential [1,2]. Unfortunately, approximately 40% of the world’s potentially cultivable land exhibits a pH level below 5.5 [2,3,4]. Under Al toxicity stress, the first and most important symptom of crops is the inhibition of root elongation, ultimately affecting water and nutrient uptake [5,6,7,8]. The high-producing area of *B. napus*, one of the important oil crops, is the Yangtze River region in China. However, the soil in this area is mainly acidic soils (pH < 5.5), and the exchangeable Al ions were precipitated from the soil to form Al toxicity stress, which limits *B. napus* growth and yield [9,10,11]. Al toxicity has emerged as a significant factor impeding crop growth in these acidic soils.

In the long-term adaption of plants to Al toxicity, plants have indeed developed two main detoxification mechanisms: external exclusion and internal tolerance [12,13]. Exposed to Al toxicity stress, the root system of the plant was initially compromised, and the root cell wall was the primary line of defense by absorbing Al ions (about 30%~90% of Al ion absorbed) or secreting organic acids to chelate Al ions, thereby performing a crucial detoxification function [12,14]. It is the complex regulatory mechanism of plant response to Al toxicity, which is regulated by multiple genes and pathways. For instance, the ANAC017-XTH31 (xyloglucan endo-transglycosylases/hydrolase 31) model regulated the Al tolerance in *Arabidopsis* by adjusting the xiylogulcan content [15]. *SbXTH7*, acting downstream of SbHY5, regulated the Al tolerance of sweet sorghum by modulating cell wall hemicellulose content [16]. F-box proteins RAE1 (AtALMT1 expression 1) and RAH1 (RAE1 homolog) affect plant resistance to Al toxicity by regulating ubiquitination and the degradation of Al core transcription factor STOP1, while ALR1 (LRR receptor-like kinase) could reduce the degradation of STOP1 by increasing the content of reactive oxygen species (ROS) in plants and inhibit the interaction between RAE1 and STOP1, thus activating the secretion of organic acid anions for detoxification [17,18,19]. In *B. napus*, a few homologous genes were cloned and verified to improve the Al tolerance of transgenic plants [20,21,22]. The Al tolerance of plants is a complex trait, which requires further exploration of new genes.

In our previous study, 64 candidate genes showed differential expression at least in 6 h vs. 0 h or 24 h vs. 0 h of two lines by integration of genome-wide association analysis (GWAS) and RNA-seq analysis [23]. With the diversification of gene mapping methods, the efficacy of bulked segregant analysis sequencing (BSA-seq) in identifying trait-associated genes has been demonstrated through extensive application in various crops [24,25,26,27]. Based on combined BSA-seq and RNA-seq analysis, 32 candidate differentially expressed genes (DEGs) response to cadmium stress in *B. napus* were identified within nine significant mapping intervals, including genes encoding a glutathione S-transferase (GST), a molecular chaperone (DnaJ), and a phosphoglycerate kinase (PGK), among others [28]. Moreover, several candidate genes associated with nitrogen utilization efficiency (NUtE) in rapeseed were screened by integration of BSA-Seq and RNA-Seq analysis, such as the high-affinity nitrate transporter gene NRT2.1 (*BnaC08g43370D*) and the abscisic acid (ABA) signal transduction related genes (*BnaC02g14540D*, *BnaA03g20760D*, and *BnaA05g01330D*); overexpression of *BnaA5.AIB* could reduce the NUtE under low N levels in *Arabidopsis* [25].

In this study, a population of F_2_ generation was constructed by crossing the Al-tolerant inbred line FDH188 (R178) and the Al-sensitive inbred line FDH152 (S169). The representative type F_2:3_ Al-tolerance was identified to select the extreme population, and then BSA-seq was employed to re-sequence the whole genome of parents and two extreme phenotypic progeny mixing pools. SNP markers were utilized for locating the target trait association region. Then, the differentially expressed genes associated with Al toxicity stress were identified in QTLs by combination with our previous transcriptome sequencing. The aim of this study was to explore Al-resistant genes in rapeseed and to provide new insights into the molecular mechanism of Al-resistant rapeseed.

## 2. Result

### 2.1. Statistical Analysis of the Phenotype of the F_2:3_ Generation Population

**Figure 1:**
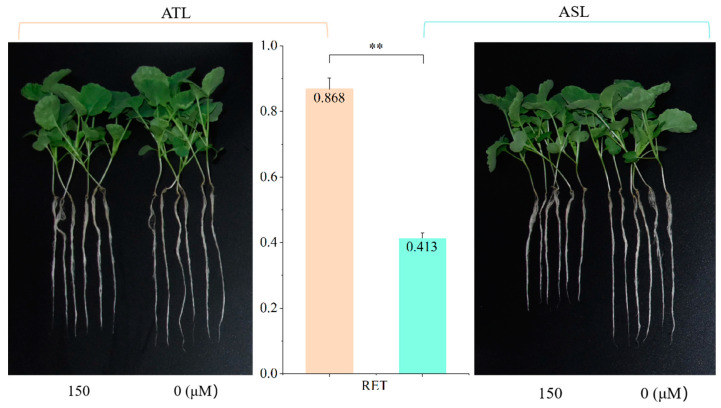
The hydroponic phenotypic of R178 (ATL) and S169 (ASL) under Al toxicity stress. Asterisks indicate significant differences between ATL and ASL (*t* test, ** *p* < 0.01).

**Figure 2:**
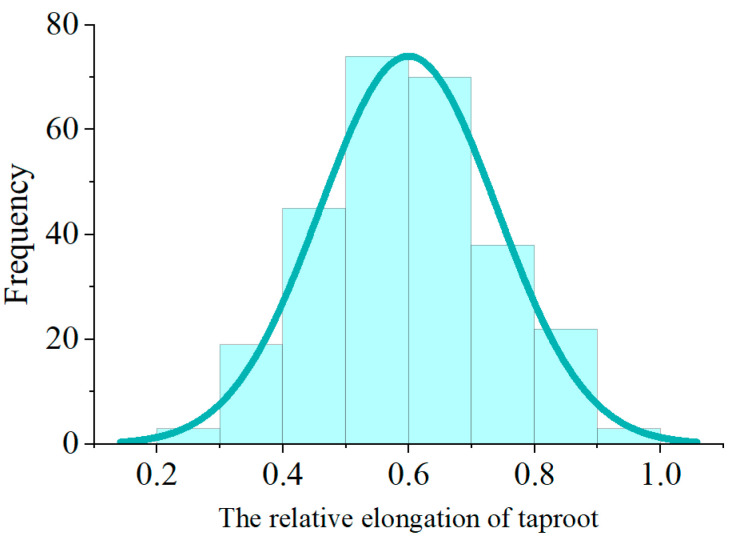
The frequency distribution of RET of the F_2:3_ population under Al toxicity stress.

After seven days of Al toxicity stress, the RET of R178 and S169 were 0.868 and 0.413, respectively (Figure 1). The phenotypic data of the F_2:3_ population showed that the F_2:3_ population exhibited maximum, minimum, and average relative elongation of taproot of 0.930, 0.281, and 0.600, and the kurtosis was −0.304; Shapiro–Wilk and Kolmogorov Smirnov tests conform to normal distribution (Figure 2 and Table 1). The results indicated that RET in the F_2:3_ population exhibits an unimodal continuous distribution without an obvious proportional relationship, exhibiting the genetic characteristics as a quantitative trait. Therefore, it is subject to polygenic regulation and suitable for QTL analysis using BSA-seq.

### 2.2. Assessment of Sequencing Quality, SNP Detection, and Annotation

A total of 1.0 billion high-quality clean reads with GC content ranging from 37.91% to 38.56% were generated (Supplementary Table S1). After the clean reads mapped to reference genome *Brassica napus* V.1, the results showed that the average sequencing depth was higher than 29 (Supplementary Table S2).

**Figure 3:**
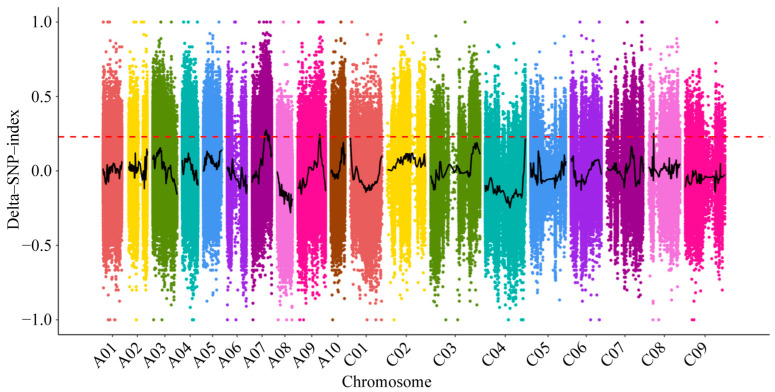
SNP-index distribution map on the whole genome. The black line is for the delta-SNP-index curve and the red line is the threshold at 99% confidence interval.

The SNP analysis results showed that 2,978,096, 3,297,428, 4,220,595, and 4,127,362 SNPs were detected in the R178, S169, ATL pool, and ASL pool, respectively. The proportion of SNP within the intergenic was found to be the highest, accounting for no less than 70%, while the number of SNP associated with splice site donors was observed to be the lowest (Supplementary Table S3). With the utilization of SNP markers Δ(SNP-index), regression fitting is performed. After fitting, when the confidence level of the delta SNP-index reaches 99% confidence interval, four QTLs are located outside the confidence interval, including A07 (15,402,793~18,799,754, 20,308,564~20,760,352), A09 (26,382,178~27,159,317), and C08 (6,061,199~6,099,270) (Figure 3, Supplementary Table S4).

### 2.3. Indel Detection and Annotation

The Indel analysis results showed that 1,004,146, 1,025,346, 1,060,849, and 1,054,800 Indels were detected in the R178, S169, ATL pool, and ASL pool, respectively. The proportion of Indel mutation sites within the intergenic was found to be the highest, accounting for no less than 65%, while the number of Indel mutation sites associated with exon deletions and stop losses was observed to be the lowest (Supplementary Table S5).

**Figure 4:**
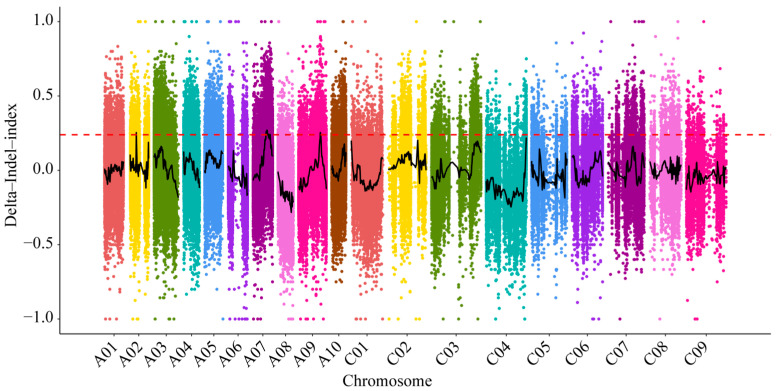
The Indel-index distribution map on the whole genome. The black line is for the delta-Indel-index curve and the red line is the threshold at 99% confidence interval.

With the utilization of SNP markers Δ(Indel-index), regression fitting is performed. After fitting, when the confidence level of the delta Indel-index reaches 99% confidence interval, four QTLs are located outside the confidence interval, including A02 (9,219,187~9,219,189), A07 (15,712,254~19,360,892, 2,068,093~20,755,053), and A09 (26,393,183~27,036,238) (Figure 4, Supplementary Table S6).

### 2.4. QTL for Al-Tolerance Identified by SNP and Indel Markers

Based on the integration of SNP and Indel markers, three QTLs on two chromosomes (A07 and A09) were identified with the confidence of Δ(SNP-index) and Δ(Indel-index) > 99%, containing *qAT-A07-1* (15,712,254~18,799,754), *qAT-A07-2* (20,680,931~20,755,053), and *qAT-A09-2* (3,125,541~3,153,126) (Table 2). Among these QTLs, *qAT-A07-1* is the longest. A total of 656 candidate genes distributed in three QTLs were located outside the 99% confidence interval, 554 candidate genes were distributed in two QTLs on chr.A07, 542 candidate genes in *qAT-A07-1*, and 102 candidate genes were distributed in one QTL on chr.A09 (Table 2, Supplementary Table S7).

### 2.5. Candidate Differentially Expresssed Genes (DEGs) Analysis

For RNA-seq, compared with 0 h, there were 2618 DEGs both at 6 h in R178 and 6 h/24 h in S169, and 576 DEGs both at 24 h in R178 and 6 h/24 h in S169 [23]. In addition, there were 8279 DEGs in R178 6 h vs. S169 6 h (4484 up-regulated and 3795 down-regulated) and 8776 in R178 24 h vs. S169 24 h (4330 up-regulated and 4446 down-regulated), including 4481 DEGs both in R178 6 h vs. S169 6 h and R178 24 h vs. S169 24 h (2108 DEGs both up-regulated, 2366 DEGs both down-regulated, four DEGs up-regulated at 6 h and down-regulated at 24 h, and three DEGs down-regulated at 6 h and up-regulated at 24 h) (Supplementary Figure S1). The combined analysis of BSA-seq and our previous RNA-seq showed that 55 candidate genes were differentially expressed, including 11 candidate DEGs both at 6 h in R178 and 6 h/24 h in S169, six candidate DEGs both at 24 h in R178 and 6 h/24 h in S169, and 45 candidate DEGs both in R178 6 h vs. S169 6 h and R178 24 h vs. S169 24 h (Table 3).

There were 46 candidate DEGs in *qAT-A07-1*, two candidate DEGs in *qAT-A07-2*, and seven candidate DEGs in *qAT-A09-1* (Table 3). Among these candidate genes, 10 genes showed differential expressions at 6 h vs. 0 h both in R178 and S169 and two genes at 24 h vs. 0 h both in R178 and S169. At 6 h vs. 0 h, six candidate genes were up-regulated both in R178 and S169, with four candidate genes both being down-regulated. At 24 h vs. 0 h, one candidate gene was up-regulated both in R178 and S169 and one gene was both down-regulated. Among R178 6 h vs. S169 6 h and R178 24 h vs. S169 24 h, 21 candidate genes were both up-regulated, 23 candidate genes were both down-regulated, and one gene was down-regulated in R178 6 h vs. S169 6 h and up-regulated in R178 24 h vs. S169 24 h. Following gene functional annotation and classification of the 55 candidate DEGs, 48 DEGs were found to be of known function, including genes encoding ABC transporter G (ABCG), zinc finger protein, NAC and ethylene-responsive transcription factor (ERF), etc., and seven DEGs uncharacterized protein.

## 3. Discussion

Al toxicity inhibited root elongation of crop and significantly decreased the relative elongation of taproot (RET) [12,16,23], which suggests that RET, as one of the main traits, was used for QTLs under Al toxicity stress [29,30]. There were 13 new candidate regions related with the relative root elongation and 69 new candidate genes response to Al toxicity in candidate regions detected in rice [30]; two QTL (*qAl_Gm13* and *qAl_Gm20*) were significantly associated with primary root length ration and may have helped maintain root elongation and initiation in soybean [29]. In our previous study, 43 SNPs significantly associated with eight Al-tolerance traits in the seedling stage were detected by GWAS, and 64 candidate genes from the flanking 100 kb region of these SNPs were screened by integration of GWAS and RNA-seq analysis [23]. During germination of rapeseed, eight SNPs (located on chromosomes A03, A07, A09, A10, C05, C06, and C09) and five SNPs (located on chromosomes A03, A04, A10, C05, and C07) were detected as significantly associated with relative root length and relative dry weight under Al toxicity [31]. In addition, 23 QTLs for five traits in rape germination period detected, including 4 QTLs related with relative root length [32], and 44 DEGs were screened by integrating the results of RNA-seq and QTL mapping of root-related traits at germination stage under Al toxicity [33]. In this study, RET of R178 (0.868) were higher than S169 (0.413) under Al toxicity stress (Figure 1). Based on the integration of SNP and Indel markers, three QTLs on two chromosomes (A07 and A09) with RET were identified between ATL and ASL, including *qAT-A07-1*, *qAT-A07-2*, and *qAT-A09-1* (Table 2). Compared with previous studies [23,31,32], three QTLs detected in current research were the novel discovered loci. And among these QTLs, a total of 55 candidate DEGs were detected by combining BSA-seq with RNA-seq, including 48 DEGs with known function and seven uncharacterized function.

In the complex process of detoxifying Al toxicity, there are many transcription factors involved in regulating the response of crops to Al stress [34,35,36,37]. The zinc finger protein STOP1 regulated plant response to Al toxicity by increasing the expression of MATE and ALMT, resulting in increasing the organic acid transported for detoxification [38,39]. OsWRKY22 enhances the expression level of *OsFRDL4*, thus improving Al-induced citrate secretion and Al tolerance in rice [40]. Both CML24 and CAMTA2 interacted with WRKY46 and alleviated the transcriptional repression of ALMT1 by WRKY46, which increased malate secretion and enhanced the Al-tolerance of the plant [36]. NAC transcription factors *VnNAR1* and *ANAC017* regulated Al tolerance by regulating the cell wall metabolism [41,42]. Moreover, over-expression of *GmABR1* (ERF transcription factor) and *GsERF1* significantly increased Al resistance in *Arabidopsis* and decreased the content of Al ions in the root tips [43,44]. In this study, seven transcription factors in *qAT*-A07-1 were detected by combining BSA-seq with RNA-seq (Table 3). Two candidate DEGs (*BnaA07g20030D* and *BnaA07g21640D*) related to zinc finger proteins showed down-regulation in R178 (6 h vs. 0 h and 24 h vs. 0 h), R178 6 h vs. S169 6 h and R178 24 h vs. S169 24 h, respectively. NAC transcription factor 29 (*BnaA07g24270D*) showed down-regulation in S169 (6 h vs. 0 h and 24 h vs. 0 h), R178 6 h vs. S169 6 h and R178 24 h vs. S169 24 h. The probable WRKY transcription factor 57 (*BnaA07g24310D*) showed down-regulation in R178 (6 h vs. 0 h and 24 h vs. 0 h) and S169 (6 h vs. 0 h). The results showed that these candidate transcription factors play an important regulatory role in *B. napus* response to Al toxicity, but the regulatory mechanism needs to be further verified.

Under Al toxicity stress, many membrane transporters play vital regulatory roles in the process of plant detoxification. For instance, genes related to the encoding Al-activated malate transporter (SgALMT2, BnALMT1, etc.) and multidrug and toxic compound extrusion (GmMATE, ZmMATE1, etc.) increased organic acid secretion in the roots to chelate Al ions, which participate in the detoxification process [21,45,46,47]. Moreover, ATP-binding cassette (ABC) transporter proteins, which are also involved in the transport and response of abacisic acid (ABA) and regulate the activity of glucosyltransferase/hydrolase in xyloglucan to affect the cell wall, play an important role in the growth and response to abiotic stress [48,49,50]. OsSTAR1 (for sensitive to Al rhizotoxicity 1) and OsSTAR2 as an ABC transporter may be used to modify the cell wall by transporting UDP-glucose for detoxification of Al in rice [50]. Knockout of *OsABCG36* resulted in increased cadmium accumulation in root cell sap and enhanced cadmium sensitivity [51]. In this study, one ABC transporter G family member 25 (*BnaA07g23320D*) in *qAT*-A07-1 was detected by combining BSA-seq with RNA-seq. The expression of *BnaA07g23320D* was both down-regulated in R178 and S169 under 6 h vs. 0 h and down-regulated in R178 6 h vs. S169 6 h and R178 24 h vs. S169 24 h. AtABCG25 is an exporter of ABA and is involved in the intercellular ABA signaling response to environmental stress among plant cells [49]. In addition, the exogenous application of ABA could increase the activity of citrate synthase and decrease Al accumulation [52,53]. In addition to ABA, Al^3+^-induced ethylene production is likely to act as a signal to alter auxin distribution in roots, which leads to local auxin accumulation in the root–apex transition zone and the arrest of root elongation [54,55,56]. Phosphoethanolamine N-methyltransferase 1 (PEAMT1) was an important regulator of root development by affecting ROS over-accumulation or inhibition of auxin signaling under choline starvation [57]. In this study, one candidate DEG (*BnaA07g23650D*) encoding the ethylene-responsive transcription factor was screened and showed up-regulation in R178 6 h vs. S169 6 h and R178 24 h vs. S169 24 h. One candidate DEG (*BnaA07g22620D*) encoding PEAMT was up-regulated at 6 h in R178 and 24 h in S169 compared with 0 h and was up-regulated in R178 6 h vs. S169 6 h. These results showed that the Al toxicity stress-induced DEGs related to the ABC transporter and ethylene-responsive transcription factor, which may affect auxin accumulation in the root–apex and inhibit root elongation.

## 4. Materials and Methods

### 4.1. Plant Materials

The Al-tolerant inbred line (FDH188, R178) and the Al-sensitive inbred line (FDH152, S169) were obtained from the Key Laboratory of Crop Physiology, Ecology, and Genetic Breeding of the Ministry of Education at Jiangxi Agricultural University [23]. R178 was selected as the male parent and S169 as the female parent; 274 F_2_ individual populations were used in this study.

### 4.2. Phenotypic Identification of Parents and F_2:3_ Population

Seeds of R178, S169, and F_2:3_ populations with full and uniform were sown on gauze in a square plastic bowl filled with pure water. After six days, the uniform and healthy seedlings were sequentially transferred into 1/4 and 1/2 Hoagland’s nutrient solution (Coolaibo Technology, Beijing, China), and each nutrient solution was cultured for three days under 25 °C-14 h/20 °C-10 h (day/night). Subsequently, the seedlings were transplanted into 0.5 mmol·L^−1^ CaCl_2_ solution (pH4.5) for 24 h. Finally, the seedlings were exposed to the 1/2 nutrient solution containing 150 µmol·L^−1^ AlCl_3_ and 0.5 mmol·L^−1^ CaCl_2_ for seven days of Al toxicity stress, with 0 µmol·L^−1^ AlCl_3_ as the control. The length of the taproot was measured both at the beginning of treatment and seven days after treatment. The relative elongation of taproot (RET) was calculated: the relative elongation of taproot = (treatment root elongation/control root elongation). The resulting data were analyzed using Excel and Origin to identify outliers within the F_2_ generation population. Three biological replicates were performed.

### 4.3. Construction of DNA Mixed Pool and Re-Sequencing

The genomic DNA of parental materials (R178, S169) and extreme individuals of the F_2_ generation population were extracted from young leaves by the CTAB method. Then, the pool of Al tolerance lines (ATL pool) was constructed by equally mixing the DNA from 27 Al-tolerance individuals. A pool of Al-sensitive lines (ASL pool) was constructed by equally mixing the DNA from 27 Al-sensitive individuals. After qualifying the DNA of R178, S169, ATL pool, and ASL pool, whole-genome re-sequencing was performed using the Illumina HiSeq platform (Genepioneer Biotechnology Co., Ltd., Nanjing, China). Then, the quality of the original reads was assessed, and the cleaned reads from four pools were compared to the reference genome sequence of *Brassica napus* V.1 by BWA software (0.7.17-r1188) [58]. Based on GATK (3.7-0-gcfedb67) [59] and SnpEff (v4_5covid19) [60], the SNPs and Indels were identified and annotated based on the comparison results.

### 4.4. Correlation Region Analysis of BSA

The Euclidean distance method (ED) is utilized to calculate the Euclidean distances between mixtures, with greater distances indicating larger differences. Regression fitting is performed on SNP marker Δ(SNP-index) and Indel marker Δ(Indel-index) located on the same chromosome to obtain a correlation threshold. The region above the 99% confidence interval of this threshold is selected as the correlation region related to the target trait.

### 4.5. Combined Analysis of BSA-Seq and RNA-Seq

For RNA-seq, R178 and S169 were treated with 150 µmol·L^−1^ AlCl_3_ for 0 h (control), 6 h, and 24 h, respectively, which has been described in our previous research [23]. To detect the DEGs, compared with 0 h, the genes with a false discovery rate ≤ 0.05 and |log_2_(fold change)| ≥ 1.0 were determined as the significantly differential expression under 6 h or 24 h Al toxicity stress in R178 and S169. In addition, the genes with a false discovery rate ≤ 0.05 and |log_2_(fold change)| ≥ 1.0 of 6 h in R178 vs. S169 or 24 h in R178 vs. S169 were considered the significantly differentially expressed genes.

After analyzing overlapping regions of SNP marker Δ(SNP-index) and Indel marker Δ(Indel-index), the candidate genes in overlapping regions were obtained. Then, combined with the results of previous RNA-seq under Al toxicity stress [23], the candidate genes with differential expression were screened. The function of candidate DEG was annotated using NR (ftp://ftp.ncbi.nih.gov/blast/db/FASTA/ (accessed on 25 March 2023)), SwissProt (http://www.expasy.org/sprot/ (accessed on 26 March 2023)).

## 5. Conclusions

In summary, three QTLs (*qAT-A07-1*, *qAT-A07-2*, and *qAT-A09-1*) were identified with the confidence of Δ(SNP-index) and Δ(Indel-index) > 99%, and 656 candidate genes were screened among these QTLs. By combining the analysis of BSA-seq and our previous RNA-seq, 55 candidate genes showed differential expression, including 11 candidate DEGs both at 6 h in R178 and 6 h/24 h in S169, 6 candidate DEGs both at 24 h in R178 and 6 h/24 h in S169, and 45 candidate DEGs both in R178 6 h vs. S169 6 h and R178 24 h vs. S169 24 h. In total, 48 out of 55 candidate DEGs with known function were detected, including genes encoding ABC transporter G (ABCG), zinc finger protein, NAC, ERF, etc. These candidate DEGs may play an important regulatory role in the *B. napus* response to Al toxicity. The combined analysis of BSA-seq and RNA-seq provided an effective strategy to explore the QTLs and candidate genes, which was useful in understanding the molecular mechanisms response to Al toxicity stress in *B. napus*.

## Figures and Tables

**Table 1:** Statistic analysis and normal distribution test of RET in the F_2:3_ population.

Indexes	F_2_ Number	Average	Minimum	Median	Maximum	Standard Deviation	CV/%	Kurtosis	Shapiro–Wilk	Kolmogorov Smirnov
Value	274	0.600	0.281	0.594	0.930	0.141	23.49	−0.304	*p* = 0.144	1.000

**Table 2:** The SNP and Indel overlapping linked regions and genes.

Chromosome	QTL	Start	End	Size/Mb	Genes
chrA07	*qAT-A07-1*	15,712,254	18,799,754	3.09	542
chrA07	*qAT-A07-2*	20,680,931	20,755,053	0.07	12
chrA09	*qAT-A09-1*	26,393,183	27,036,238	0.64	102

**Table 3:** The candidate DEGs related Al-resistance screened by BSA-seq and RNA-seq.

Gene ID	QTL	Function Description	R178		S169		R178 vs. S169	
			6 h vs. 0 h	24 h vs. 0 h	6 h vs. 0 h	24 h vs. 0 h	6 h	24 h
*BnaA07g20010D*	*qAT-A07-1*	DeSI-like protein At4g17486	−1.43	-	−1.59	-	1.59	-
*BnaA07g20030D*	*qAT-A07-1*	Zinc finger protein 1	−1.15	−1.42	−1.08	-	-	-
*BnaA07g20140D*	*qAT-A07-1*	Vacuolar fusion protein CCZ1 homolog B	-	-	-	-	−1.67	−1.48
*BnaA07g20150D*	*qAT-A07-1*	Chaperone protein dnaJ 8, chloroplastic	-	-	-	-	−2.52	−2.49
*BnaA07g20260D*	*qAT-A07-1*	Protein ACTIVITY OF BC1 COMPLEX KINASE 3, chloroplastic	-	-	-	-	3.15	1.46
*BnaA07g20290D*	*qAT-A07-1*	Phosphoglycerate kinase 3, cytosolic	-	-	-	-	−1.71	−1.48
*BnaA07g20500D*	*qAT-A07-1*	DNA repair protein recA homolog 1, chloroplastic	-	-	-	-	1.69	1.90
*BnaA07g20510D*	*qAT-A07-1*	Photosystem II 10 kDa polypeptide, chloroplastic	-	-	−1.89	-	3.05	1.17
*BnaA07g20530D*	*qAT-A07-1*	Interactor of constitutive active ROPs 4	1.08	-	1.28	-	-	-
*BnaA07g20740D*	*qAT-A07-1*	Protein COFACTOR ASSEMBLY OF COMPLEX C SUBUNIT B CCB4, chloroplastic	-	-	-	-	−10.51	−8.94
*BnaA07g20870D*	*qAT-A07-1*	Thaumatin-like protein	-	-	-	-	1.76	1.69
*BnaA07g20960D*	*qAT-A07-1*	Indole glucosinolate O-methyltransferase 1	-	1.22	-	1.19	1.89	1.53
*BnaA07g21010D*	*qAT-A07-1*	Ubinuclein-2	-	-	-	-	−1.82	−2.43
*BnaA07g21360D*	*qAT-A07-1*	Increased DNA methylation 3	-	-	-	-	−1.15	−1.22
*BnaA07g21490D*	*qAT-A07-1*	Dehydrin ERD14	-	−2.26	-	−2.19	−1.10	-
*BnaA07g21500D*	*qAT-A07-1*	PREDICTED: pro-resilin-like [Brassica rapa]	-	-	-	-	−1.16	−1.08
*BnaA07g21560D*	*qAT-A07-1*	Rac-like GTP-binding protein ARAC5	-	-	-	-	2.72	2.71
*BnaA07g21640D*	*qAT-A07-1*	B-box zinc finger protein 21	-	-	-	-	−1.47	−1.70
*BnaA07g21850D*	*qAT-A07-1*	Glycine--tRNA ligase, chloroplastic/mitochondrial 2	-	-	-	-	−1.52	−1.33
*BnaA07g21970D*	*qAT-A07-1*	Protein MARD1	2.01	-	2.13	-	-	-
*BnaA07g22050D*	*qAT-A07-1*	Protein NUCLEAR FUSION DEFECTIVE 4	-	-	1.35	-	−1.59	−1.28
*BnaA07g22100D*	*qAT-A07-1*	Probable arabinosyltransferase ARAD1	-	-	-	-	−1.58	−1.77
*BnaA07g22220D*	*qAT-A07-1*	GDSL esterase/lipase At1g74460	-	-	-	-	1.48	1.79
*BnaA07g22620D*	*qAT-A07-1*	Phosphoethanolamine N-methyltransferase 3	1.63	-	-	4.73	5.71	-
*BnaA07g22680D*	*qAT-A07-1*	Sucrose synthase 6	-	-	−3.04	-	3.61	2.44
*BnaA07g22710D*	*qAT-A07-1*	protein-lysine methyltransferase METTL21D [Brassica napus]	-	-	-	-	−3.16	−5.01
*BnaA07g22740D*	*qAT-A07-1*	Serine carboxypeptidase-like 2	-	-	−1.40	-	−2.22	−3.67
*BnaA07g22770D*	*qAT-A07-1*	F-box/LRR-repeat protein At5g02910	-	-	-	-	−3.62	−2.69
*BnaA07g22810D*	*qAT-A07-1*	Anaphase-promoting complex subunit 13	-	-	-	-	2.52	2.25
*BnaA07g22890D*	*qAT-A07-1*	Beta-galactosidase 17	-	-	-	2.94	3.79	2.07
*BnaA07g23200D*	*qAT-A07-1*	Protein NRT1/ PTR FAMILY 5.11	-	-	-	-	2.25	2.37
*BnaA07g23230D*	*qAT-A07-1*	Protein NRT1/ PTR FAMILY 5.11	1.85	-	1.81	-	1.35	2.23
*BnaA07g23320D*	*qAT-A07-1*	ABC transporter G family member 25	−1.59	-	−1.59	-	−2.57	−2.51
*BnaA07g23350D*	*qAT-A07-1*	Sucrose transport protein SUC1	−2.21	-	−2.07	−1.15	−4.03	−3.87
*BnaA07g23650D*	*qAT-A07-1*	Ethylene-responsive transcription factor ERF070	-	-	-	-	1.05	1.58
*BnaA07g23760D*	*qAT-A07-1*	Probable peroxygenase 4	-	-	-	-	−2.39	−2.67
*BnaA07g24010D*	*qAT-A07-1*	Transcription factor KUA1	-	−1.58	−1.14	-	-	−1.44
*BnaA07g24230D*	*qAT-A07-1*	Cyclic dof factor 5	−5.40	−1.09	−6.03	-	-	-
*BnaA07g24270D*	*qAT-A07-1*	NAC transcription factor 29	-	-	−1.05	−1.53	−1.20	−4.43
*BnaA07g24310D*	*qAT-A07-1*	Probable WRKY transcription factor 57	−1.16	−1.48	−2.11	-	-	-
*BnaA07g28730D*	*qAT-A07-2*	Protein ALTERED XYL	-	-	-	-	1.08	1.02
*BnaA07g28790D*	*qAT-A07-2*	Probable peroxygenase 4	-	-	-	-	−3.11	−3.13
*BnaA09g36740D*	*qAT-A09-1*	Pentatricopeptide repeat-containing protein At3g57430, chloroplastic	-	-	-	-	−1.30	−1.27
*BnaA09g36900D*	*qAT-A09-1*	protein enabled homolog [*Brassica napus*]	-	-	-	-	1.10	1.19
*BnaA09g36940D*	*qAT-A09-1*	Probable glucuronoxylan glucuronosyltransferase F8H	-	-	−1.79	-	2.29	1.48
*BnaA09g37240D*	*qAT-A09-1*	E3 ubiquitin-protein ligase SINAT2	-	-	-	-	1.13	1.08
*BnaA09g37360D*	*qAT-A09-1*	MATH domain and coiled-coil domain-containing protein At3g58340	-	-	-	-	2.71	2.18
*BnaA09g37400D*	*qAT-A09-1*	Rhomboid-like protein 15	-	-	-	-	1.66	1.40
*BnaA07g20780D*	*qAT-A07-1*	PREDICTED: uncharacterized protein LOC103830546	1.38	-	1.14	-	-	-
*BnaA07g21140D*	*qAT-A07-1*	uncharacterized protein	-	-	-	-	−2.03	−1.75
*BnaA07g21190D*	*qAT-A07-1*	uncharacterized protein	-	-	-	-	−9.48	−8.96
*BnaA07g23870D*	*qAT-A07-1*	PREDICTED: uncharacterized protein LOC103830885 [*Brassica rapa*]	-	-	-	-	1.72	1.37
*BnaA07g24050D*	*qAT-A07-1*	uncharacterized protein	−1.62	−2.01	-	-	−1.01	−1.24
*BnaA07g24110D*	*qAT-A07-1*	PREDICTED: uncharacterized protein LOC103830913 [*Brassica rapa*]	−2.36	-	-	-	−2.76	2.66
*BnaA09g36790D*	*qAT-A09-1*	uncharacterized protein BNAA09G36790D [*Brassica napus*]	-	-	-	-	3.05	1.99

## Data Availability

The data presented in this study are available in this article and Supplementary Materials.

## Supplementary Materials

The following supporting information can be downloaded at https://www.mdpi.com/article/10.3390/ijms252011190/s1.
