# Supplementary figures and images for: Combined BSA-Seq and RNA-Seq Analysis to Identify Candidate Genes Associated with Aluminum Toxicity in Rapeseed (Brassica napus L.)
Source: Int J Mol Sci. 2024 Oct 17;25(20):11190. doi: 10.3390/ijms252011190 (PMC11514608; doi:10.3390/ijms252011190)

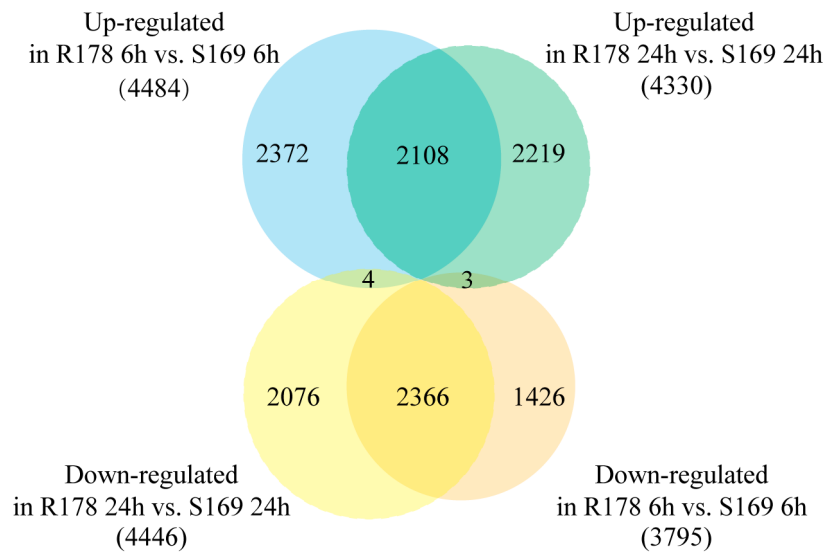

Supplementary Figure S1 The DEGs in R178 6h vs. S169 6h and 178 24h vs. S169 24h

Supplement: Supplementary file 1 [file ijms-25-11190-s001.zip › Supplementary Figure S1.pdf]
